# Supplementary material for: Distinct functions of three Wnt proteins control mirror-symmetric organogenesis in the C. elegans gonad
Source: eLife. 2024 Nov 1;13:e103035. doi: 10.7554/eLife.103035 (PMC11620738; doi:10.7554/eLife.103035)
Supplement: Supplementary file 1. [file elife-103035-supp1.docx]

Supplementary File 1. List of strains used for experiments

| Strain Name | Genotype |
| --- | --- |
| HS2663 | *lin-17(n3091) ; tkIs12(mig-24::Venus)* |
| HS1720 | *mom-5(ne12)/hT2[qIs48]* |
| HS1403 | *lin-17(n3091) mom-5(ne12)/hT2[qIs48]* |
| HS3321 | *lin-17(n3091) mom-5(ne12)/hT2[qIs48] ; osIs113(lin-17p::LIN-17 ΔCRD::GFP65c) ; tkIs12(mig-24::Venus)* |
| HS1747 | *lin-17(os2) mom-5(ne12)/hT2[qIs48]* |
| HS2710 | *lin-17(mn589) mom-5(ne12)/hT2[qIs48]* |
| CF314 | *mig-1(e1787) lin-17(n671)* |
| HS1164 | *lin-17(n3091) ; cfz-2(ok1201)* |
| HS1402 | *lin-17(n3091)/hT2[qIs48] ; lin-18(e620)* |
| HS2092 | *lin-17(n3091) ; cam-1(gm122) ; vpIs1(elt-3::GFP)* |
| HS2552 | *lin-17(n3091) ; cwn-1(ok546) ; vpIs1(elt-3::GFP)* |
| HS2635 | *lin-17(n3091) ; cwn-2(ok895) ; tkIs12(mig-24::Venus)* |
| HS4215 | *lin-17(n671) ; cwn-2(ok895) ; tkIs12(mig-24::Venus)* |
| HS3323 | *lin-17(n3091) ; cwn-2(ok895) ; osIs113(lin-17p::LIN-17 ΔCRD::GFP65c) ; tkIs12(mig-24::Venus)* |
| HS2533 | *lin-17(n3091) ; egl-20(n585) ; vpIs1(elt-3::GFP)* |
| HS2617 | *lin-17(n3091) ; egl-20(n585) cwn-2(ok895) ; tkIs12(mig-24::Venus)* |
| HS2844 | *lin-17(n3091) ; cwn-1(ok546) ; egl-20(n585) ; tkIs12(mig-24::Venus)* |
| HS2603 | *lin-17(n3091)/hT2[qIs48] ; cwn-1(ok546) ; cwn-2(ok895) ; tkIs12(mig-24::Venus)* |
| HS2866 | *lin-17(n3091) ; cwn-1(ok546) ; egl-20(n585) cwn-2(ok895)/nT1[qIs51] ; tkIs12(mig-24::Venus)* |
| HS2873 | *lin-17(n3091) ; cwn-1(ok546) ; egl-20(n585) cwn-2(ok895)/nT1[qIs51] ; tkIs12(mig-24::Venus) ; osEx395(ceh-22::cwn-1::wVenus)* |
| HS2982 | *lin-17(n3091) ; cwn-1(ok546) ; egl-20(n585) cwn-2(ok895)/nT1[qIs51] ; tkIs12(mig-24::Venus) osIs93(egl-20p::cwn-2::wVenus)* |
| HS2675 | *mom-5(ne12)/hT2 [qIs48] ; cwn-1(ok546) ; egl-20(n585) cwn-2(ok895) ; vpIs1(elt-3::GFP)* |
| HS1263 | *qIs74(GFP::POP-1)* |
| HS1112 | *lin-17(n3091) ; qIs74(GFP::POP-1)* |
| HS1768 | *mom-5(ne12)/hT2 [qIs48] ; qIs74(GFP::POP-1)* |
| HS2453 | *lin-17(n3091) mom-5(ne12)/hT2[qIs48] ; qIs74(GFP::POP-1)* |
| HS2630 | *lin-17(n3091) ; cwn-2(ok895) ; qIs74(GFP::POP-1)* |
| HS2667 | *lin-17(n3091) ; egl-20(n585) cwn-2(ok895) ; qIs74(GFP::POP-1)* |
| HS2623 | *lin-17(n3091)/hT2 [qIs48] ; cwn-1(ok546) ; cwn-2(ok895) ; qIs74(GFP::POP-1)* |
| HS3110 | *lin-17(n3091) ; cwn-1(ok546) ; egl-20(n585) cwn-2(ok895)/nT1 [qIs51] ; qIs74(GFP::POP-1)* |
| JK3930 | *qIs95(sys-1p::VENUS::SYS-1)* |
| HS2753 | *lin-17(n3091) ; qIs95(sys-1p::VENUS::SYS-1) ; cwn-2(ok895)* |
| HS2754 | *lin-17(n3091) ; qIs95(sys-1p::VENUS::SYS-1) ; egl-20(n585) cwn-2(ok895)* |
| HS2749 | *lin-17(n3091) ; egl-20(n585) cwn-2(ok895) ; tkIs12(mig-24::Venus) ; osEx402(egl-20p::cwn-2::wVenus)* |
| HS3642 | *lin-17(n3091) ; egl-20(n585) cwn-2(ok895) ; osIs168(lag-2p-mkikGR)* |
| HS3987 | *xnSi1(mex-5::GFP::PH::nos2 3'UTR) ; qyIs127(lam-1::mcherry) ; mes-1(bn7ts) ; osEx283(mig-24p::Venus)* |
| HS3988 | *lin-17(n3091) ; xnSi1(mex-5::GFP::PH::nos2 3'UTR); egl-20(n585) cwn-2(ok895) ; qyIs127(lam-1::mcherry) ; tkIs12(mig-24::Venus)* |
| HS2483 | *unc-76(e911) ; osEx443(sys-1p::GFP(NLS))* |
| FX1422 | *vang-1(tm1422)* |
| HS2637 | *cwn-1(ok546) II ; cwn-2(ok895) IV ; vang-1(tm1422)* |
